# Bilateral benign renal oncocytomas and the role of renal biopsy: single institution review

**DOI:** 10.1186/s12894-016-0190-2

**Published:** 2017-01-12

**Authors:** Andrew R. Leone, Laura C. Kidd, Gregory J. Diorio, Kamran Zargar-Shoshtari, Pranav Sharma, Wade J. Sexton, Philippe E. Spiess

**Affiliations:** Department of Genitourinary Oncology, H. Lee Moffitt Cancer Center and Research Institute, 12902 Magnolia Drive Office 12538, Tampa, FL 33612 USA

**Keywords:** Bilateral, Biopsy, Oncocytoma, Renal cell carcinoma, Synchronous

## Abstract

**Background:**

The goal was to assess the natural history and management of patients with pathologically proven bilateral (synchronous) RO after undergoing initial partial nephrectomy (PN).

**Methods:**

All patients underwent either robotic/laparoscopic or open PN by two experienced genitourinary oncologists from 2005–2013. Final pathology was determined by surgical excision, CT-guided percutaneous core biopsy (CT-biopsy) or fine needle aspiration (FNA). Patient demographics, tumor characteristics (pathologic data, location, size) type of surgery, pre/post estimated glomerular filtration rate (eGFR) and surgical complications were recorded.

**Results:**

Twelve patients were identified with bilateral RO. Median age at the time of surgery was 68 years (46–77) (Table 1). The median size of the largest tumor(s) resected was 2.75 cm (1.5–5.5 cm) and second largest tumor(s) was 1.75 cm (1.0–4.0 cm). Four patients underwent bilateral staged PN and one patient underwent simultaneous bilateral PN (horseshoe kidney). Two patients underwent RFA at the time of biopsy of the contralateral mass after PN. Five patients underwent CT-bx/FNA (5/5) of the contralateral mass followed by active surveillance.

Mean follow up was 34 months. There was no significant change in median creatinine pre- and post-operatively. One patient was lost to follow up and one patient died of unknown causes 5 years post-operatively. eGFR decreased an average of 16.96% post-operatively, including a single patient whose eGFR increased by 7.8% after surgery and a single patient whose eGFR did not change (Table 2).

**Conclusions:**

Patients with bilateral renal masses and pathologically proven RO can be safely managed with active surveillance after biopsy confirmation of the contralateral mass.

## Background

Renal oncocytoma (RO) is the second most common benign renal neoplasm which arises from the intercalated collecting duct cells in the kidney [1, 2]. It has an estimated incidence of 3 to 7% of all primary renal masses [3, 4] and comprises 10 to 15% of enhancing small renal masses (≤4 cm in diameter) [5]. It commonly presents in men in the seventh decade of life and is often diagnosed incidentally [3]. RO can be unilateral or bilateral (4 to 14% of cases), solitary or multifocal (2 to 12% of cases) and can exist concurrently with malignant components, specifically renal cell carcinoma (RCC) [6], frequently chromophobe RCC (chRCC) [7]. Oncocytosis has been used to describe numerous discrete oncocytic nodules, usually with the presence of a larger primary nodule [7].

In clinical practice, RO often represents a diagnostic challenge, due to its similarity in appearance to renal cell carcinoma on both pathology and imaging, [2] specifically chromophobe RCC. RO and chromophobe RCC share a common cellular origin from intercalated cells [8]. Recent research has attempted to distinguish the two tumors. The radiologic finding of “segmental enhancement inversion” on computerized tomography (CT) has shown acceptable specificity, but tends to be reliable in a size-dependent matter [2]. Also, this finding has shown low sensitivity, ranging from 15 to 21% in magnetic resonance imaging (MRI) and CT respectively, and only fair inter-observer agreement [9]. One recent study showed a high sensitivity (receiver-operating characteristic area under the curve 0.817, *p* < 0.001) of predicting RO versus chRCC with the combined presence of a central stellate scar and higher Hounsfield Units (HUs) on multiphase CT [8]. Despite these and other studies, there is an inability to clinically accurately distinguish between RCC and RO based on imaging alone. Thus, enhancing suspicious small renal masses (SRMs) are treated as probable malignancy and managed via surgical excision (partial or radical nephrectomy (RN)), ablation, or surveillance [10, 11]. Recently, the role of renal mass biopsy (RMB) has experienced resurgence in popularity in managing patients with SRMs. In an effort to avoid the morbidity associated with treatment modality, and possibly avoid overtreatment in patients with bilateral renal masses, some have even advocated for all patients with SRM to undergo RMB preoperatively [12].

The efficacy and practical role of biopsy in the diagnosis of renal oncocytoma has been a focus of study in recent years. There have been variable data to both support and refute the use of a core or FNA prior to deciding upon management. Depending on the adequacy of the tissue obtained, pathology can result as either a specific subtype of RCC, RO, or a mix of both, called hybrid oncocytic/chromophobe tumors (HOCT). When pathologists cannot discriminate between chRCC and oncocytoma, the designation of “oncocytic neoplasm” is used [13]. Again, this contributes to a clinical dilemma in how to manage these patients.

Despite the risk of concurrent malignancy, however, there is a shift toward more conservative management of SRMs. This study sought to investigate the use of renal biopsy following unilateral partial nephrectomy in the setting of primary bilateral synchronous renal oncocytomas. This subset has been previously observed in a case series of three patients in 2010 [14]. Our cohort of patients represents the largest presenting with bilateral renal oncocytomas. We propose that an approach involving partial nephrectomy proven diagnosis of oncocytoma with bilateral masses undergo renal mass biopsy. In the case of confirmation of oncocytoma or oncocytic neoplasm, patients can be safely followed on active surveillance protocols.

## Methods

After institutional review board approval MCC 15666, we searched our nephrectomy database between 2005 and 2013 for all patients with bilateral oncocytoma. Twelve patients were identified who had also undergone either a robotic/laparoscopic or open partial nephrectomy by two surgeons (PES, WJS) at Moffitt Cancer Center in Tampa, Florida. Further record provided the final pathology, which was determined by surgical specimen and correlated with CT-guided percutaneous core biopsy or fine needle aspirate. Size of biopsy needle, as well as biopsy technique was variable and performed at the discretion of the radiologist. Additional patient information was gathered retrospectively, including: age, gender, BMI, largest and next largest tumor sizes, number of tumors per patient, pre-operative creatinine, the presence of absence of pre-operative chronic kidney disease (CKD), post-operative creatinine at one month, percent of patients surviving to follow-up, median follow-up time and percent undergoing radiofrequency ablation (RFA). Management decisions were based on surgeon discretion, based on factors such as linear growth rate kinetics, size of the contralateral tumor, patient comorbidities and patient preference.

Patient demographic data was listed and pathological characteristics were reported based on phrasing used in the patients’ charts. Renal function pre- and post-operatively was also recorded. Estimated GFR (eGFR) was calculated via the CKD-EPI equation with CKD classifications per KDIGO practice guidelines [15]. Follow-up was determined on an individual basis at the discretion of the two surgeons listed above. Patients were generally followed in clinic with labs and imaging every three to six months for two years, with increasing intervals after that. All statistics were performed using IBM SPSS software version 22.

## Results

Of the 583 patients in our database from 2005 to 2013, twelve were found who were diagnosed with synchronous bilateral renal oncocytoma and had undergone unilateral partial nephrectomy. Nine of these patients had also undergone subsequent CT-guided biopsy or FNA of the contralateral kidney. Table 1 summarizes patient demographics. The patient median age at time of surgery was 68 years (46–77). The median size of the largest tumor(s) resected was 2.75 cm (1.5–5.5 cm). Median size of the second largest tumor(s) was 1.75 cm (1.0–4.0 cm). Four patients underwent bilateral staged PN and one patient underwent simultaneous bilateral partial nephrectomy for horseshoe kidney. Two patients underwent radiofrequency ablation at the time of biopsy of the contralateral mass following PN. Five patients underwent CT-guided biopsy (*n* = 5) and FNA (*n* = 5) of the contralateral mass after PN, followed by active surveillance with routine imaging. There were no reported complications secondary to FNA or core biopsy collection. No patients required repeat intervention for suspected malignancy at a mean follow-up time of 34 months. There was no significant change in median creatinine pre- and post-operatively (0.95 mg/dL and 1.01 mg/dL, respectively). Two patients (16.7%) demonstrated CKD (defined as eGFR <60 mL/min/1.73 m^2^) pre-operatively, which doubled after the surgeries to four patients with CKD (33.3%). One patient was lost to follow up and one patient died of unknown causes five years after PN. Of the five followed by active surveillance, three showed no growth at one year via CT scan and two had minimal growth, 3 mm and 1 mm.

**Table 1:** Demographic and outcomes data

Parameter	
Number of patients	12
Mean Age (yrs.)	66.8
Gender	
Male	10
Female	2
Mean BMI (kg/m^2^)	32.1
Median largest tumor size (cm)	2.75
Median next largest tumor (cm)	1.75
Mean Pre-operative Creatinine (mg/dL)	0.95
Pre-operative CKD^b^	16.7%
Post-operative CKD^b^	33.3%
Post-operative Creatinine (mg/dL) at 1 month	1.01
Median follow-up after PN (months)	33.5
Alive at follow-up	91.7%^a^
Underwent RFA	8.3%

^a^Excludes one patient lost to follow-up

^b^CKD defined as eGFR <60 mL/min/1.73 m^2^

Estimated GFR was calculated via the CKD-EPI equation and definitions of CKD were per KDIGO [15] guidelines. Pre-operative and post-operative CKD category, creatinine (mg/dL) and eGFR are all listed in Table 2, as well as percent change in eGFR after surgery at follow-up. Three of 12 patients (25%) were at stage G1, 7 of 12 (58.3%) at stage G2 and 2 patients (16.7%) were at G3a prior to surgery. After surgery, one of the 12 patients (8.3%) was at stage G1, 7 of 12 (58.3%) were at G2, 2 of 12 (16.7%) were at G3a, likewise for G3b, with no patients falling into G4 and G5 ranges. eGFR decreased an average of 16.96%, including a single patient whose eGFR increased by 7.8% after surgery and a single patient whose eGFR did not change post-operatively.

**Table 2:** Follow-up Characteristics

Patient	Age*	Pre-op Creatinin e (mg/dL)	Pre-op eGFR^†^	Pre-op CKD (Category)^†^	Follow-up after PN (months**)	Follow-up Creatinine (mg/dL)	Follow-up eGFR^†^	Post-op CKD (Category)^†^	Change in eGFR^†^
1	55–60	0.7	104	G1	3.9	0.8	100	G1	-3.8%
2	65–70	0.8	75	G2	24.0	0.9	64	G2	-14.7%
3	65–70	1.0	77	G2	19.6	1.1	68	G2	-11.7%
4	70–75	0.8	88	G2	49.3	0.8	86	G2	-2.3%
5	65–70	0.9	87	G2	69.2*	1.6	42	G3b	-51.7%
6	60–65	0.7	102	G1	55.1	1.2	63	G2	-38.2%
7	75–80	0.9	62	G2	64.7	1.3	38	G3b	-38.7%
8	65–70	1.4	51	G3a	19.4	1.3	55	G3a	+7.8%
9	45–50	1.0	90	G1	125.3*	1.0	84	G2	-6.7%
10	65–70	1.0	79	G2	43.0	1.2	61	G2	-22.8%
11	75–80	1.3	53	G3a	2.5	1.3	53	G3a	0%
12	65–70	0.9	87	G2	0.2	1.1	69	G2	-20.7%

* range provided to ensure no identifying information

** follow-up period from first partial nephrectomy

^†^ eGFR calculated using CKD-EPI equation, definitions of CKD per KDIGO guidelines

Table 3 details the procedure performed (PN vs. RN, FNA vs. core biopsy) and laterality, surgical pathology and sizes of tumors, as well as biopsy pathology. Two patients who underwent bilateral PN, also had either FNA or core biopsy performed. In one of the two cases, initial right FNA showed “oncocytic neoplasm, favor oncocytoma”, while right PN surgical pathology confirmed oncocytoma. In the second case, left core biopsy showed “renal neoplasm with oncocytic features”, confirmed as oncocytoma after left PN, as well as right FNA demonstrative of “cytologic change consistent with oncocytoma” and right PN surgical pathology affirmed the diagnosis of oncocytoma.

**Table 3:** Pathological Characteristics

Left Kidney					Right Kidney			
Patient	# of Tumors	Tumor Sizes (cm)	Management	Pathology	# of Tumors	Tumor Sizes (cm)	Management	Pathology
1	--	--	Core bx + AS	“renal oncocytic neoplasm”	1	1.6	PN	“oncocytoma”
2	--	--	PN	“oncocytoma”	1	1.5	PN	“oncocytoma”
3	1	4.0	PN	“oncocytoma”	--	--	FNA + AS	“oncocytic neoplasm”
4	4	1.6, 1.5, 1.5, 0.7	PN	“oncocytoma”	--	--	FNA + PN	“oncocytic neoplasm (favor oncocytoma)”
5	1	4.8	PN	“oncocytoma”	1	2.0	PN	“oncocytoma”
6	--	--	Core bx + AS	“oncocytoma”	1	2.3	PN	“oncocytoma”
7	1	3.0	PN	“oncocytoma”	--	--	FNA + AS	“oncocytic neoplasm (favor oncocytoma)”
8	2	2.5, 2.0	PN	“oncocytoma”	--	--	Core bx + AS	“oncocytic neoplasm (c/w oncocytoma)”
9	6	3.1, 3.0, 2.7, 0.8, 0.4, 0.1	Core bx + PN	“oncocytoma” (surgical pathology); “renal neoplasm with oncocytic features” (biopsy)	6	5.5, 4.0, 2.8, 2.8, 2.2, 0.7	FNA + PN	“cytologic changes c/w oncocytoma”
10	4	1.5, 1.0, 1.0, 0.4	PN	“oncocytoma”	--	--	Core bx + RFA	“renal oncocytoma”
11	--	--	FNA + RFA	“cytologic changes c/w oncocytoma”	1	3.5	PN	“oncocytoma”
12	1	1.5	PN	“renal oncocytic neoplasm”	1	3.2	RN*	“oncocytoma”

*PN* partial nephrectomy; *RN radical nephrectomy (patient with horseshoe kidney); *bx* biopsy; *FNA* fine needle aspiration; *RFA* radiofrequency ablation; *AS* active surveillance

## Discussion

Despite the relative rarity and benign nature of renal oncocytoma ad compared to other renal tumors, the possibility of co-existence with RCC warrants further evaluation when suspected on incidental imaging. Although scarce, there have been few cases of development of contralateral RCC in patients with RO, with chromophobe carrying the best prognosis [7]. In the case of renal oncocytosis, besides hybrid tumors, chRCC has been shown to be both the most common malignant dominant tumor type (26%; 6 of 23 specimens) as well as secondary tumor type (70%; 16 of 53 specimens) found on histology.

Childs et al. investigated metachronous renal tumors following PN of primary RO, showing that these newly arising tumors were more likely to be benign compared to patients presenting with a newly diagnosed SRM; of 12 patients with either surgical or biopsy pathology, 8 were RO versus 4 RCC (of various types). They also determined that the risk of metachronous RCC after PN of renal oncocytoma was similar to that of the general population, and utilized a less aggressive surveillance approach with ultrasound (US) versus CT [16].

Other studies have confirmed the benign nature of oncocytoma by showing no evidence of metastasis in patients both undergoing active surveillance and/or nephrectomy. Bhatt et al. published 36 patients with RO, 34 of whom underwent nephrectomy, none of whom developed metastases at a median follow-up time of 84 months after surgery [17]. Similar results were obtained in a cohort of 20 patients with oncocytosis, who showed no metastases at a median follow-up of 35 months, although CKD was a major morbidity, with 50% affected prior to surgery and 25% developing CKD after nephrectomy [7]. One consideration in that case is the presence of multiple oncocytic nodules which have been shown to intersperse between the nephrons and affect nearby tubules in oncocytosis [7]. Familial syndromes can also play a role in the clinical and pathologic presentation of oncocytoma, which is associated with the autosomal dominant Birt-Hogg-Dubé (BHD) syndrome. The most common tumor type found in these patients is a hybrid mixed histology, containing features of both oncocytoma and RCC, [7] often termed “oncocytic neoplasms” by pathologists [13].

Recent investigation has also elucidated more about the growth rates of renal oncocytoma. These rates have been cited at 0.14 to 0.20 cm per year [5, 18], compared to small renal masses in general, at 0.28 cm per year,, according to a meta-analysis [18], and 0.38 cm/yr for chRCC [5].

Renal mass biopsy has shown to have promising utility. Diagnostic yield on first biopsy has been shown to be 80.6 to 93% [13, 19, 20] with overall improvement in yield with a subsequent biopsy [13, 19]. Core biopsy sensitivity and specificity are 99.1 and 99.7%, respectively, while FNA is 93.2 and 89.8%, respectively [19]. In addition, complications have not been found to be a major morbidity in RMB, with studies quoting complication rates between 8.5 and 10.4%, most of which were minor and self-limited [13, 20]. Both the high rate of diagnostic accuracy of RMB, as well as the low rate of complications, argues in favor of biopsy and subsequent implementation of an active surveillance protocol.

A single-institution study of 15 patients diagnosed with renal oncocytoma via CT-guided biopsy was followed to better elucidate the course of RO with watchful waiting. Mean follow-up time was 30 months and showed that 6 of the 15 underwent surgery (2 partial and 4 total nephrectomies) for rapid growth (0.5 cm/yr.), tumor burden or patient preference. All surgical pathologies were confirmed RO except for a single case of oncocytoma associated with chRCC (T2 Fuhrman grade 3). Nine of the 15 patients underwent biannual ultrasound imaging and were asymptomatic at a mean follow-up time of 49.7 months. Those who underwent surgery were younger and had a significantly higher tumor growth velocity than the non-operative patients (2.4 ± 2.1 mm/yr versus 0.7 ± 0.5 mm/yr., *P* = 0.008) [21].

This possibility of harboring malignant histology was demonstrated in a study of 20 patients with oncocytosis. After either partial or radical nephrectomies, 13 patients had dominant tumor hybrid histology, followed by 6 chRCC, 3 pure oncocytoma and 1 with clear cell RCC. Multiple secondary nodules were examined and were also varied in histology, including hybrid, chRCC, pure RO, conventional RCC and clear cell papillary RCC [7].

One essential consideration in patients diagnosed with SRMs is that of preserving renal function. In our single institution review, 2 of 12 patients had baseline CKD with 2 more developing it after surgery (bilateral PN and left PN). Lane et al. showed that medically-induced CKD (versus surgically-induced CKD) was associated with greater rate of progressive decline in renal function, all-cause mortality and non-renal cancer mortality, with non-renal mortality and GFR stability being similar in the surgically-induced CKD and non-CKD cohorts. Another study of 1928 patients showed male gender (odds ratio (OR) 3.55), older age at diagnosis of SMR (OR 1.04), hypertension (OR 0.46), serum calcium (OR 2.06) and lower serum albumin (OR 0.23) to be significantly correlated with pre-operative CKD. Additionally, radical nephrectomy, as compared to partial nephrectomy, was shown to have the strongest association with post-operative CKD (OR 11.89). Hence, the authors of that paper suggested consideration of elderly men with hypertension as candidates for PN versus radial nephrectomy to avoid development of CKD [22].

Although the latter study only addressed RCC patients, these are certainly still factors to be considered in the setting of patients with bilateral RO. Taken together, they suggest the importance of careful screening of patients for risk factors pre-operatively, which may compel intervention instead of surveillance.

In summary, renal mass biopsy followed by active surveillance has been considered safe [5, 23], reliable, cost-effective, with no change in survival overall. Use of renal mass biopsy has increased and should also be applied to the unique subset of patients suspected of having bilateral renal oncocytoma. Biopsy plus active surveillance is also of benefit because it does not preclude the patient from future urologic interventions, such as partial nephrectomy or ablation [23].

Limitations of this study include its retrospective nature and one patient lost to follow-up. We also had a small sample size, due to the rarity of bilateral synchronous oncocytoma. Our median time to follow-up was 34 months, which could perhaps not be long enough to capture development of new tumors or other complications in the disease course after partial nephrectomy. Also, we recommend the use of confirmatory biopsy in diagnosing the contralateral tumor, but not every patient in our cohort underwent biopsy and/or FNA (only 9 out of 12). Another limitation is the fact that all patients in our cohort underwent partial nephrectomy, denying us the ability to observe these masses free of intervention.

## Conclusion

From our single institution experience we believe that patients with unilateral renal oncocytoma with contralateral mass who have undergone unilateral partial nephrectomy of the RO should undergo confirmatory biopsy of the contralateral mass and can be safely managed with active surveillance imaging protocols. Future prospective multi-institutional trials are needed to determine optimal management of bilateral renal masses in the setting of prior unilateral oncocytoma.

## Abbreviations

AS: Active surveillance
chRCC: Chromophobe type, renal cell carcinoma
FNA: Fine needle aspiration
RCC: Renal cell carcinoma
RMB: Renal mass biopsy
RO: Renal Oncocytoma
SRM: Small renal mass
